# A Comparative Analysis of Aquatic and Polyethylene-Associated Antibiotic-Resistant Microbiota in the Mediterranean Sea

**DOI:** 10.3390/biology10030200

**Published:** 2021-03-06

**Authors:** Arianna Sucato, Luca Vecchioni, Dario Savoca, Alessandro Presentato, Marco Arculeo, Rosa Alduina

**Affiliations:** 1 Department of Biological, Chemical and Pharmaceutical Sciences and Technologies (STEBICEF), University of Palermo, Viale delle Scienze, Bd. 16, 90128 Palermo, Italy; sucato.arianna@gmail.com (A.S.); alessandro.presentato@unipa.it (A.P.); 2 Department of Biological, Chemical and Pharmaceutical Sciences and Technologies (STEBICEF), University of Palermo, Via Archirafi, 18, 90123 Palermo, Italy; luca.vecchioni@unipa.it (L.V.); marco.arculeo@unipa.it (M.A.); 3 Department of Biological, Chemical and Pharmaceutical Sciences and Technologies (STEBICEF), University of Palermo, Viale delle Scienze, Bd. 17, 90128 Palermo, Italy; dario.savoca@unipa.it

**Keywords:** microbiome, resistome, plastisphere, polyethylene, aquatic environments, antibiotic resistant bacteria, antibiotic resistance genes, integron

## Abstract

**Simple Summary:**

In recent years, a growing interest has been devoted to the bacterial characterization of marine plastic debris. So far, a few publications have explored the composition of microbial communities on polyethylene (PE) waste items and the occurrence of antibiotic-resistant bacteria (ARB). The occurrence of ARB in natural matrices can contribute to the spread of antibiotic resistance genes (ARGs) among environmental bacteria. In this study, we compared the microbial composition and the presence of ARGs in water and PE fragments collected from a stream and the seawater in a coastal area of Northwestern Sicily. Our findings showed more ARGs on PE fragments than the corresponding water samples, confirming that PE can act as a carrier of antibiotic-resistance genes causing high damage to the marine environment and living organisms.

**Abstract:**

In this study, we evaluated the microbiome and the resistome profile of water and fragments of polyethylene (PE) waste collected at the same time from a stream and the seawater in a coastal area of Northwestern Sicily. Although a core microbiome was determined by sequencing of the V3–V4 region of the bacterial 16S rDNA gene, quantitative differences were found among the microbial communities on PE waste and the corresponding water samples. Our findings indicated that PE waste contains a more abundant and increased core microbiome diversity than the corresponding water samples. Moreover, PCR analysis of specific antibiotic resistance genes (ARGs) showed that PE waste harbors more ARGs than the water samples. Thus, PE waste could act as a carrier of antibiotic-resistant microbiota, representing an increased danger for the marine environment and living organisms, as well.

## 1. Introduction

Plastics are currently considered as the most common and abundant form of marine debris, which is attracting particular attention for the health of both environment and living organisms. The main sources of synthetic plastics in the marine environment are represented by waste from coastal tourism, fishing, marine industries, and manufacturing of plastic products, being the release of about 12,000 Mt of plastic waste expected by 2050 [1].

Plastic waste can be accidentally ingested by animals [2,3] and edible marine species, representing a danger for human food security and health [4,5]. Besides, plastics could release chemical additives such as phthalates or bisphenols [3], and, given their hydrophobic nature, they could absorb and transport various types of pollutants (e.g., hydrocarbons, polychlorobiphenyls, and dioxins, to name a few) across ecosystems. Moreover, plastic debris could act as a suitable substrate for the development of bacterial biofilms, which can contain pathogens or antibiotic-resistant bacteria (ARB) [6]. Therefore, plastic biofilms can be regarded as a new microbial niche in the environment [7,8,9,10,11]. To date, although the high interest in the investigation of plastics in marine ecosystems, relatively little is known about the microbial composition of plastics, indicated as the “plastisphere” [9,12,13]. Plastics can be considered as a hotspot for bacterial contact facilitating the horizontal gene transfer among microbes [14] and could represent a vector for the spread of ARB or human pathogens into the marine environment [15,16]. Indeed, environmental marine bacteria, which could be already antibiotic-resistant, may become attached to marine plastic litter and be carried and dispersed via passive transport [17]. The distribution of antibiotic-resistant bacteria through plastic debris in aquatic ecosystems is underestimated. The antibacterial resistance is considered as one of the biggest public threat to wild and farm animals and human health [18,19]. The presence of antibiotics and antibiotic resistance genes (ARGs) in environmental matrices can contribute to the diffusion of resistance determinants among environmental bacteria [7,20,21,22,23,24,25,26,27,28,29,30]. Hospitals, farms, aquacultures, and wastewater treatment plants (WWTPs) are considered as “hotspot environments” of ARGs and ARB, where bacteria are exposed to high and repeated doses of antibiotics, nutrient abundance, and suitable environmental conditions [18]. Thus, antimicrobial agents and pathogenic resistant bacteria can access sewage through the waste released from these “hotspot environments”, reaching water ecosystems with the final effluent [31]. Since ARGs are frequently associated with gene cassettes containing the class 1 integron [32,33], a mobile genetic element commonly found in Gram-negative bacteria, responsible for the conjugative-mediated gene transfer [34], the concomitant presence of ARGs and the mobile element *int1* gene into environmental metagenomics DNA samples represents an alarming concern.

Although the increase in the study of the plastisphere, no agreement has been reached on whether plastic-associated communities feature an increased or decreased diversity compared with their counterparts in the water [35]. Specifically, our hypothesis was to evaluate if plastic, particularly fragments of polyethylene (PE) waste, could serve as a carrier of microbial communities and antibiotic resistance genes increasing the spread of antibiotic resistant strains into aquatic environments.

Hence, this study aims to evaluate the microbiome and the resistome profile of water and PE waste samples collected at the same time from a stream and the seawater in a delimited coastal area of Northwestern Sicily.

## 2. Materials and Methods

### 2.1. Study Area and Sample Collection

**Figure 1:**
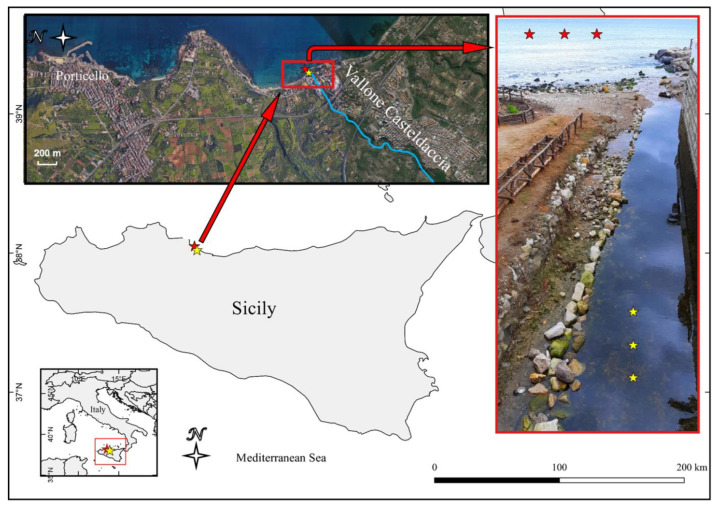
Map of the sampling site. Red asterisks indicate the sampling site of seawater and PE waste from the sea and the yellow ones the sampling site from the stream.

During a sampling campaign conducted in October 2019, three PE waste samples were collected on the bottom of a small stream, named Vallone Casteldaccia, located on Northwestern Sicily—(Italy, 38°03′52.69″ N; 013°32′16.35″ E) together with three water samples from different close spots of the same stream. All samples were taken in a range of approximately 5 m at a depth of about 20 cm. The map of the sampling sites (Figure 1) was created using the QGIS software v.2.18.2 (http://www.qgis.org, accessed on 19 January 2021). These samples are indicated as “freshwater PE” (FP) and “freshwater” (FW). Additionally, three PE waste samples, indicated as “seawater PE” (SP) and four “seawater” (SW) samples were collected in front of the stream near the coastline (20 m off) at a depth of 1.5 m. All plastic samples consisted of 5–10 cm fragments of PE waste while the water samples had a volume of one liter. PE waste and water samples were collected and stored in one-liter sterile glass containers, placed in the dark in an icebox until transport to the laboratory.

### 2.2. DNA Extraction, PCR Amplification, and Sequencing

Total DNA extraction was carried out using the method already described [36] with a few minor changes. Individual small pieces of PE waste (of the same size as the 50 mL tube stopper) were sorted with sterile tweezers and rinsed twice using sterile distilled water before the DNA extraction. Of water samples 5 mL were used to extract metagenomics DNAs. The purity and quantity of DNA were assessed via spectrophotometry (Nanodrop, Thermo Fisher Scientific, Waltham, MA, USA). DNA was quantified and an equal amount of template DNA of each PE waste and water sample was used (10 ng). The extracted DNA was used to amplify a 464 bp fragment corresponding to the V3-V4 region of the 16S rDNA using the primers described in Takahashi et al. [37], and amplicons were sent to BMR Genomics srl, Padova (PD) for DNA sequencing in one 300-bp paired-end run on an Illumina MiSeq platform.

Raw sequences were imported to the QIIME2 environment [38] (https://qiime2.org, accessed on 10 September 2020) as paired-end sequences. DADA2 plug-in was used in order to filter, remove chimeras, and to denoise all our sequences in order to obtain the OTU (operation taxonomic units). Taxonomy was assigned, from each OTU, using the SINA classifier on the latest SILVA dataset available [39] (https://www.arb-silva.de/ngs/, accessed on 11 September 2020). Rarefaction analysis was carried out plotting the number of observed OTUs against the total number of filtered reads for each sample. The differences in terms of abundances among the studied samples (SP, SW, FP, and FW) were analyzed by ANOVA one-way using the statistical software MINITAB 17. In addition, in order to evaluate the variations among samples, Principal Coordinate Analysis (PCoA) was performed using the software package PRIMER 6 [40]. The analyses were based on Bray–Curtis distance matrix. Diversity indices were performed as described in Arizza et al. [36]. Sequences were deposited in GenBank (BioProject PRJNA662461).

### 2.3. Detection of Antibiotic Resistance Genes (ARGs)

Of the metagenomic DNA 10 ng was utilized as a template to amplify genes coding for products responsible for the resistance to antimicrobials, such as erythromycin *ermB*, tetracycline *tetA* and *tetW*, sulfonamides *sulII*, β-lactams *blaTEM* and *blaCTXM,* and quinolones *qnrS*. Moreover, the *int1* gene was investigated. All PCR reactions were performed using the annealing temperature and the primer pairs listed in Table 1. The presence of the expected amplification product was considered as a positive sample. The primers reported in Table 1 were used to control DNA quality to amplify a 142 bp DNA fragment of the 16S rDNA gene (Table 1).

## 3. Results

### 3.1. Microbiome Sequencing Output and Analysis

In total, 466,246 high-quality reads (Q > 33 and 470 bp in size) were filtered from 658,687 raw reads obtained from thirteen samples. Using the QIIME2 pipeline 3264 unique operational taxonomic units (OTUs) were successfully identified (Table 2) and classified at the family level using a 97% sequence similarity threshold against the “Silva” database.

Unassigned OTUs were categorized as “Unclassified”. The estimation of rarefaction curves indicated a satisfactory level of diversity sampling (Supplementary Figure S1). Good’s coverage (Table 3), which estimates the completeness of sampling, showed a high level (0.959–0.996) in the identification of bacterial groups, except for sample FW3 (0.901), that was excluded for further analysis since a low number of total reads (444) were obtained. 

Interestingly, an average of 554 ± 128.9 OTUs was obtained in PE waste collected from seawater (SP), while the corresponding seawater (SW) samples contained almost three times fewer OTUs (198 ± 22.5). A less pronounced difference in OTUs was evidenced between FW (219 ± 21.2) and the corresponding PE waste (256 ± 99), although the latter featured a slightly higher amount. The mean number of OTUs and the coefficient of variation of each sample indicated a greater variability among PE waste samples than water ones (Table 4).

Bacterial diversity estimated by the Shannon–Wiener index varied from 2.88 to 4.04 except sample FW3 (Table 4), which, as previously mentioned, was not taken into consideration. Simpson index and evenness showed no significant difference between samples. Furthermore, abundance-based richness estimators, Chao1 and ACE, showed several phylotypes ranging from 129 to 288 (Table 4). ANOVA (one-way) analysis showed that the four groups were significantly different with a *p*-value of 0.002 (Table 5).

### 3.2. Taxonomic Composition

**Figure 2:**
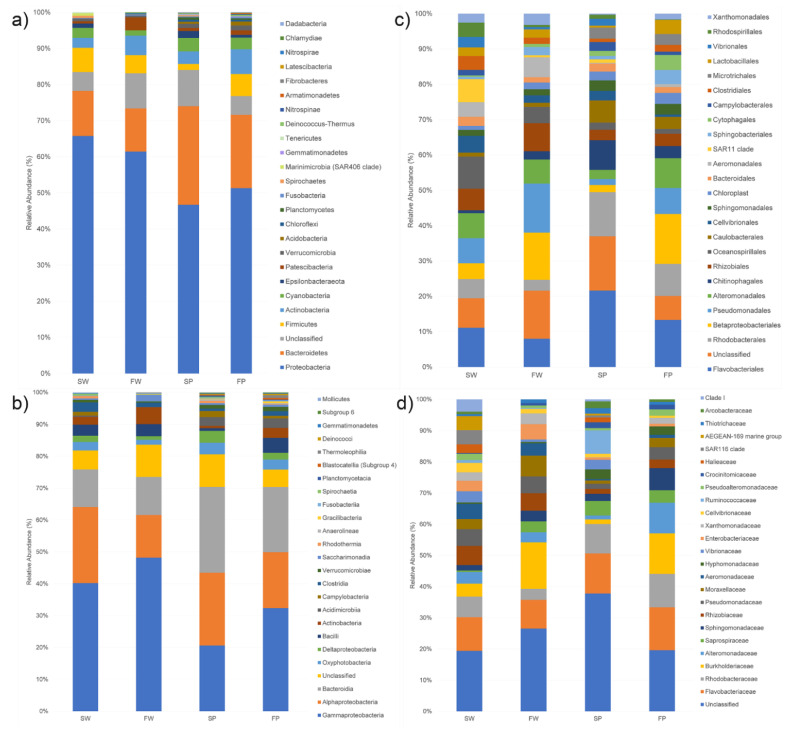
Bar plots reporting the relative percentage abundance of the bacterial phyla (**a**), classes (**b**), orders (**c**), and families (**d**) in seawater (SW), freshwater (FW), and the corresponding PE wastes (SP and FP). Each bar represents the mean of the grouped samples. Microbial composition was determined taking into account only the 25 most abundant taxa.

The taxonomic analysis of all samples yielded a total of 28 phyla, 57 classes, 142 orders, and 248 families. Fragments of PE waste featured a more diverse microbial community than the corresponding water samples, indeed SP and FP samples contained 24 and 20 phyla respectively, while 14 and 12 phyla were identified in SW and FW samples, respectively (Figure 2). Proteobacteria was the most dominant phylum in SW (66%) and FW (61%), while in SP and FP this phylum accounted for 47% and 51%, respectively (Figure 2a). The second most dominant phylum was represented by Bacteroidetes with 12% in SW and FW samples, 28% and 20% in SP and FP, respectively. Although Firmicutes was the phylum less represented among the dominant ones, its presence prevailed in both water samples and in FP. Actinobacteria and Patescibacteria were more represented in freshwater samples than seawater ones. Besides, Chlamydiae were found only in the freshwater sample, while Dadabacteria, Elusimicrobia, and Hydrogenedentes were associated solely with seawater PE.

Among classes, Gammaproteobacteria was the most dominant in the SW (40%), FW (47%), and FP (32%) samples (Figure 2b). Differently, in SP, Bacteroidia was the most represented class (26%) followed by Alphaproteobacteria (23%) and Gammaproteobacteria (20%). In SW and FW, Alphaproteobacteria were also abundant (24 and 13%) followed by Bacteroidia (12%), while in FP Bacteroidia (20%) were more abundant than Alphaproteobacteria (17%).

Among orders (Figure 2c), Flavobacteriales was the most abundant in SW (8%) and both PE samples (17 and 10% in SP and FP, respectively), while it was less abundant in FW (6%). SW samples were mainly characterized by Oceanospirillales (7%), Pseudomonadales (5%), Alteromonadales (5%), SAR11 clade (5%), Rhizobiales (5%), and Rhodobacterales (4%). FW contained an equal percentage of Betaproteobacteriales and Pseudomonadales (ca. 10%) as dominant components, followed by Flavobacteriales (6%), Rhizobiales (6%) Alteromonadales (5%), and Aeromonadales (4%). SP was mostly characterized by Rhodobacterales (10%), Chitinophagales (6%), and Caulobacterales (5%). FP contained, besides Flavobacteriales, Betaproteobacteriales (11%), Rhodobacterales (7%), Alteromonadales (7%), and Pseudomonadales (6%). At the family level, all samples contained different percentages of Flavobacteriaceae (5.6–8.7%), Rhodobacteraceae (2.1–6.8%), and Burkholderiaceae (0.9–9.1%), while Vibrionaceae were mainly found in SW and SP (Figure 2d).

**Figure 3:**
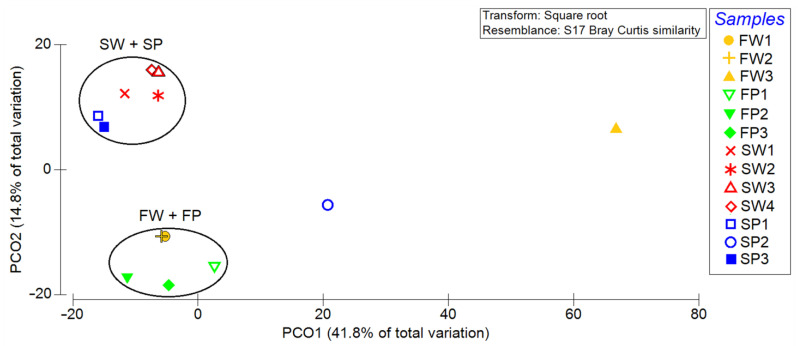
Principal coordinate analysis (PCoA) plot highlighting the clustering of microbiome samples. SW, FW, SP, and FP stand for seawater, freshwater, seawater PE, and freshwater PE, respectively. Samples FW3 and SP2 are not reported.

The principal coordinate analysis (PCoA) plot based on the Bray–Curtis distance matrix showed that the four samples formed two clusters, one consisting of PE waste (blue symbols in Figure 3) and water (red symbols) collected from the sea, and the other containing PE waste and water from the freshwater. Only samples FW3 (removed from previous analysis) and SP2 can be considered as outliers (Figure 3).

### 3.3. Resistome Analysis

**Figure 4:**
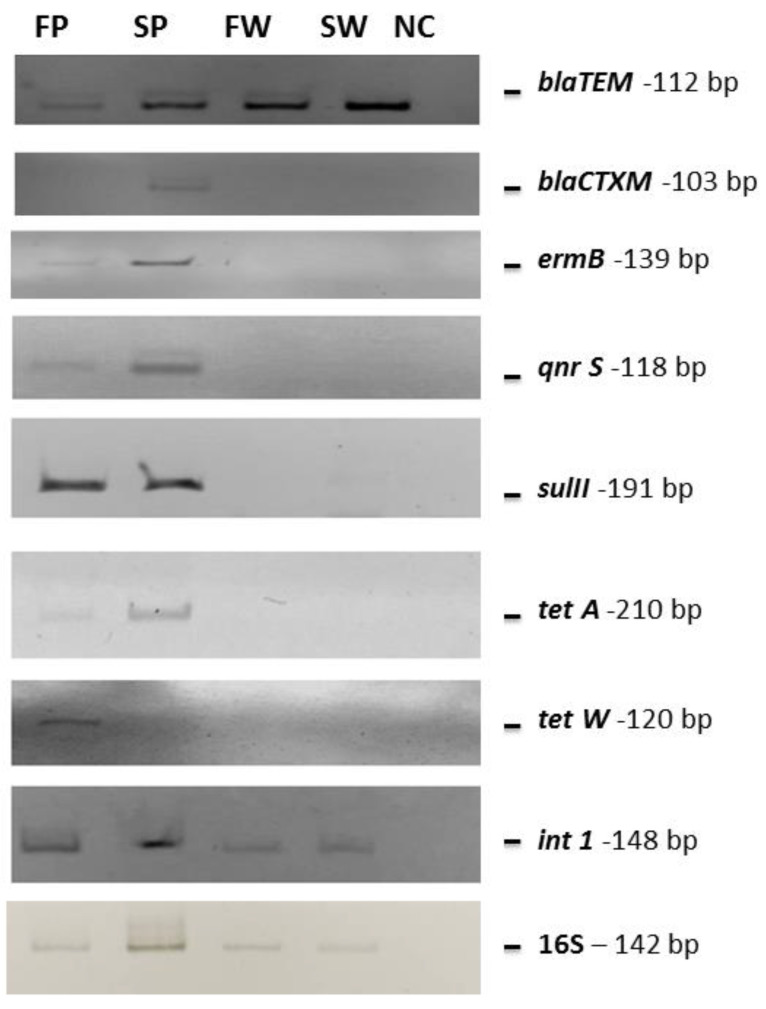
PCR gels showing the obtained amplified bands. The same quantity of pooled DNA (10 ng) as the template was used in each reaction. SW, FW, SP, and FP stand for seawater, freshwater, seawater PE, and freshwater PE, respectively. NC indicates the negative control (water instead of DNA as template).

Metagenomic DNAs deriving from each sample was pooled and analyzed by PCR for *bla*_TEM_, *bla*_CTXM_, *ermB*, *qnrS*, *sulII*, *tetA,* and *tetW* genes, which are very common antibiotic-resistance determinants in the Mediterranean Sea [20,21,22,23,25,26]. All samples were positive for the presence of the *bla*_TEM_ gene, responsible for β-lactam resistance, while the *bla*_CTXM_ gene was detected only in the PE waste collected from the seawater. The *ermB*, *qnrS*, *sulII*, and *tetA* genes were detected only in the PE waste samples collected from both seawater and freshwater, while the *tetW* one was found only in freshwater PE waste. Moreover, the mobile element *int1* gene was amplified from all analyzed samples (Table 6 and Figure 4).

## 4. Discussion

In this study, we show that the analyzed fragments of PE waste were richer in bacterial diversity and ARGs than the corresponding water samples in which waste was dispersed. This aspect confirms that the sampling area has an important role in determining the bacterial assemblage, as already suggested elsewhere [35]. This difference could be attributable to different environmental conditions (salinity, temperature, pH, etc.) or a diverse period of persistence of PE waste into the water, while freshwater and seawater could have more stable conditions and a less quantity of nutrients (N, P, etc.). Our findings revealed a higher number of OTUs in PE waste collected from both seawater and freshwater than in the corresponding seawater and freshwater samples (Table 3), suggesting that PE wastes represent an aquatic bacteria-enriched habitat acting as a good substrate for bacterial colonization. Our results agreed with a recent published study in which bacteria were found to be associated with substrates made of PE [48].

Proteobacteria, Bacteroidetes, and Firmicutes represent the dominant phyla in all the samples, ranging from 76 to 83% of the total bacteria. Actinobacteria and Patescibacteria were more represented in freshwater collected samples than in the seawater ones, suggesting their origin from the soil. Recently, Actinobacteria, known as prolific antibiotic producer soil bacteria, are being isolated from freshwater and this is becoming an emerging area in the field of microbiology [49], while Patescibacteria were found to preferentially flourishing under oligotrophic conditions [50]. Chlamydiae, known as human obligate intracellular pathogens, were found only in one freshwater sample [51], while some phyla, i.e., Dadabacteria and Hydrogenedentes, only in seawater PE wastes. Our results confirm those obtained by recent studies on Dadabacteria, considered as cosmopolitan bacteria of the marine environment [52] and Hydrogenedentes, assumed as putative organic carbon degraders, potentially hydrolyzing carbon compounds such as phthalates, of which plastics are made of [53].

Alphaproteobacteria, Gammaproteobacteria, and Bacteroidia represented homogeneously the dominant microbial classes contributing over 69% of the total microbial communities, accordingly with the results reported by Tu et al., 2020 [48] that consider them as the core microbiome of the PE-associated biofilms. The Alphaproteobacteria class was more represented in seawater-collected samples as compared to freshwater ones. This class includes the SAR11 clade, also known as Pelagibacterales, found more abundant in seawater than the other samples. The members of this order are believed to play an important role in the mineralization of dissolved organic carbon and are implicated in the uptake of phosphate, an important process in the oligotrophic zones since phosphorus is also a limiting nutrient in seawater [54].

Flavobacteriaceae, found in all the samples, represent the major component of bacterioplankton, abundant in marine environments [55]. The Rhodobacteraceae family was more abundant in PE waste samples since its members are identified as the primary colonizers of surfaces during the earliest stages of the biofilm formation [56]. Lastly, the Burkholderiaceae family, more abundant in freshwater samples, includes some Gram-negative pathogens, which are generally found in soils or untreated surface waters [57].

The possibility of finding antibiotic-resistant bacteria in marine waters is now well documented and attributable to the excessive use of antibiotics in the healthcare and farm [19]; these reach usually the sea through wastewater or simply from the river. In the present study, the *bla_TEM_* gene was found in all analyzed samples, and the β-lactams resistance was frequently observed in the marine ecosystem [19,21,25,26]. The resistance to β-lactam antibiotics was frequently found in seawater, fishes, and wild marine species, like sea turtles, which could be involved in the spread of this resistance [20,58,59]. Differently, the *bla*_CTXM_ gene was found only in seawater PE wastes and was not determined in a previous study carried out using sea water samples [20]. *tetA* and *sulII* genes were found solely in PE waste samples, differently from other works attesting their prevalent presence in surface water [60,61].

The *int1* gene was found in all the samples, indicating a warning for the spread of ARB and ARGs, as already indicated elsewhere [19]. Furthermore, the frequency of class I integrons has been postulated as an indicator of anthropogenic pollution in the environment [62]. Indeed, the widespread presence of the *int1* gene in our samples highlights the potential transfer of ARGs between different bacterial strains and their migration between connected aquatic systems. Their diffusion into marine environments would increase the risk to human health because of the ineffectiveness of antibiotics for treating infectious bacterial diseases [61].

Moreover, we found an increased number of ARGs in samples collected from both seawater and freshwater PE wastes, which contain six of the seven analyzed genes. We hypothesized that water from the freshwater contains ARB and ARGs that can be absorbed on the PE wastes and transported along the streamline to the sea, where PE wastes can stay longer and can become concentrators of microbes. PE wastes collected from freshwater also contained a high number of ARGs, indicating the negative anthropogenic role in water contamination. Seawater and freshwater contained only *blaTEM* and *int1* genes, while freshwater PE wastes contained the *tetW* gene. Tetracyclines are commonly used in both the treatment of human infections and livestock production, for example swine and cattle farms [22,23,63]. The *tetW* gene was detected, as an example, in a river catchment of the Pearl River in China, which is heavily influenced by human activities [61].

Another important source of antibiotic resistance, often overlooked, comes from mariculture (floating cages) in which the operators used fishmeal made of contaminated animal species [64]. Indeed, resistance to antibiotics could be acquired by wild marine organisms directly by polluted water or through unconsumed food during feeding of farmed species (usually sea bass, sea bream, or salmon) that settles, accumulating in the substrate or dispersed in the water column. All these residual food particles move into the food chain, finally reaching man as final consumer. Thus, the antibiotic resistance can be acquired through the consumption of contaminated wild or farm meat or by direct contact with the seawater. This is the case, for example, of the antibiotic-resistance found in edible marine species such as fish and mollusks found along the polluted coasts of Campania [65] or in farmed species such as cows, pigs, chickens, fish, etc. [64]. Overall, special attention must be focused on the area where mariculture plants or intensive cattle or poultry farms are present.

The exceeding presence of ca. 250,000 tons [53] of plastic constitute another possible carrier of ARGs. This represents an alarming aspect concerning the marine pollution caused by this debris since the actual amount of plastic is strongly underestimated due to the absence of microplastics in the above-reported quantification [54]. It is now well known that plastic (macro and micro) is of particular concern for the environment, and for the health of animals and people; in fact, they can determine negative effects on the marine biota and, indirectly, also on humans. Plastics are not only a problem linked to their direct, albeit accidental, ingestion as it occurs, for example, in turtles [3,66,67] or other vertebrates or marine invertebrates [68].

Our results demonstrated that PE wastes collected from seawater were richer in bacterial diversity and ARGs that could be passively transported through the sea streamlines. Thus, we confirm, in line with recent studies [6,17], that PE wastes could represent a reservoir for antibiotic resistance contributing to disseminating resistant bacteria in the seawater.

## 5. Conclusions 

In this study, we described the microbial community occurring in the water and that colonizing the PE fragments collected from freshwater and the adjacent seawater. Our results demonstrated that the plastisphere featured richer microbial biodiversity than the corresponding water. Besides, the plastisphere contained a higher number of antibiotic-resistant genes than the corresponding water samples, indicating that the plastics could represent an alarming threat to the marine environment and living organisms, considering plastic waste as contributing to the dispersal of bacteria and antibiotic resistance determinants.

## Figures and Tables

**Table 1:** List of primers used in this study.

Target Name	Primer Sequence (5′-3′)	Amplicon Size (bp)	Annealing Temperature (°C)	Reference
*16S rDNA for V3-V4 sequencing*	cctacgggnbgcascag	464	55	[37]
	gactacnvgggtatctaatcc			
*16S rDNA*	cggtgaatacgttcycgg	142	55	[32]
	gghtaccttgttacgactt			
*tetA*	gctacatcctgcttgccttc	210	64	[41]
	catagatcgccgtgaagagg			
*bla_TEM_*	ttcctgtttttgctcacccag	112	60	[42]
	ctcaaggatcttaccgctgttg			
*bla_CTXM_*	ctatggcaccaccaacgata	103	60	[43]
	acggctttctgccttaggtt			
*qnrS*	gacgtgctaacttgcgtgat	118	62	[44]
	tggcattgttggaaacttg			
*sulII*	tccggtggaggccggtatctgg	191	60	[45]
	cgggaatgccatctgccttgag			
*ermB*	ccgaacactagggttgctc	139	55	[33]
	atctggaacatctgtggtatg			
*tetW*	acatcattgatactccaggtcacg	120	56	[46]
	tttcactttgtggttgaacccctc			
*int1*	ggcttcgtgatgcctgctt	148	59	[47]
	cattcctggccgtggttct			

**Table 2:** Total number of operational taxonomic units (OTUs) resulting from the QIIME2 pipeline dataset.

Tag *	Total Reads	Merged Reads	Filtered Reads	Chimeras	OTUs
SP1	26,675	16,644	18,340	270	592
SP2	111,375	80,453	67,895	6599	410
SP3	47,278	23,472	37,537	1212	659
SW1	85,593	52,885	60,866	8000	211
SW2	80,494	55,120	60,922	6563	213
SW3	73,051	45,744	53,042	8863	204
SW4	6414	4040	4471	127	165
FP1	8556	5250	5401	18	144
FP2	51,174	26,681	40,353	5480	331
FP3	43,526	25,192	34,954	5000	293
FW1	47,811	29,800	34,770	2281	204
FW2	69,353	40,831	47,251	4153	234
FW3	7387	142	444	0	14

* SW stands for seawater samples, FW for freshwater, SP for PE collected from seawater, and FP for PE collected from freshwater.

**Table 3:** Average number and coefficient of variation of OTUs detected in water and PE waste collected from freshwater and seawater.

Sample	Mean ± Std.Dev	C.v. OTUs
Seawater (SW1-SW4)	198 ± 22.5	11.3
Freshwater (FW1-FW2)	219 ± 21.2	9.7
Seawater PE (SP1-SP3)	554 ± 128.9	23.3
Freshwater PE (FP1-FP3)	256 ± 99	79.2

**Table 4:** Diversity indexes of the studied samples. S is the total number of families; Chao1 and ACE are abundance-based richness estimators; α is the alpha diversity; 1-D is the Simpson’s index; H’ is the Shannon–Wiener diversity; e is the evenness.

Sample	S	Good’s Coverage	Chao1	ACE	α	1-D	H’	e
SP1	146	0.96	285.32	281.79	4.05	0.05	4.04	0.81
SP2	53	0.99	288.36	286.13	7.71	0.1	2.88	0.72
SP3	119	0.97	202.39	205.51	5.53	0.07	3.46	0.72
SW1	64	0.99	264.68	260.92	3.29	0.03	3.68	0.88
SW2	64	0.99	274.20	269.82	3.25	0.04	3.59	0.86
SW3	67	0.99	276.49	274.66	3	0.02	3.76	0.89
SW4	75	0.96	288.09	284.83	2.20	0.04	3.81	0.88
FP1	53	0.97	280.71	278.42	2.71	0.04	3.49	0.88
FP2	81	0.99	180.05	189.58	4.08	0.06	3.49	0.79
FP3	70	0.99	227.11	227.53	4.18	0.07	3.28	0.77
FW1	69	0.99	237.77	236.87	2.96	0.04	3.63	0.85
FW2	71	0.99	251.34	248.52	3.28	0.06	3.49	0.82
FW3	13	0.90	129.16	137.30	1.07	0.02	2.41	0.95

**Table 5:** One-way ANOVA based on the samples’ abundances. Significance level α = 0.05. DF, degree of freedom; Adj SS, adjusted sum of squares; Adj MS, adjusted mean of squares; F-value, F statistic.

Source	DF	Adj SS	Adj MS	F-Value	*p*-Value
Sample	3	303,233	101,078	11.00	0.002
Error	9	82,728	9192		
Total	12	385,961			

**Table 6:** Summary of the presence/absence of ARGs and int1 gene detected in metagenomics DNA samples extracted from water and PE waste collected from freshwater and seawater.

Sample	*bla_TEM_*	*bla_CTXM_*	*ermB*	*qnrS*	*sulII*	*tetA*	*tetW*	*int1*
Seawater (SW1-SW4)	+	−	−	−	−	−	−	+
Freshwater (FW1-FW3)	+	−	−	−	−	−	−	+
Seawater PE (SP1-SP3)	+	+	+	+	+	+	−	+
Freshwater PE (FP1-FP3)	+	−	+	+	+	+	+	+

## Data Availability

The data presented in this study are available in GenBank at https://www.ncbi.nlm.nih.gov/genbank/ (BioProject PRJNA662461).

## Supplementary Materials

The following are available online at https://www.mdpi.com/2079-7737/10/3/200/s1, Figure S1: Estimation of rarefaction curves indicated a satisfactory level of diversity sampling.
