# Supplementary material for: A Comparative Analysis of Aquatic and Polyethylene-Associated Antibiotic-Resistant Microbiota in the Mediterranean Sea
Source: Biology (Basel). 2021 Mar 6;10(3):200. doi: 10.3390/biology10030200 (PMC8001005; doi:10.3390/biology10030200)
Supplement: Supplementary file 1 [file biology-10-00200-s001.pdf]

biology

MDPI

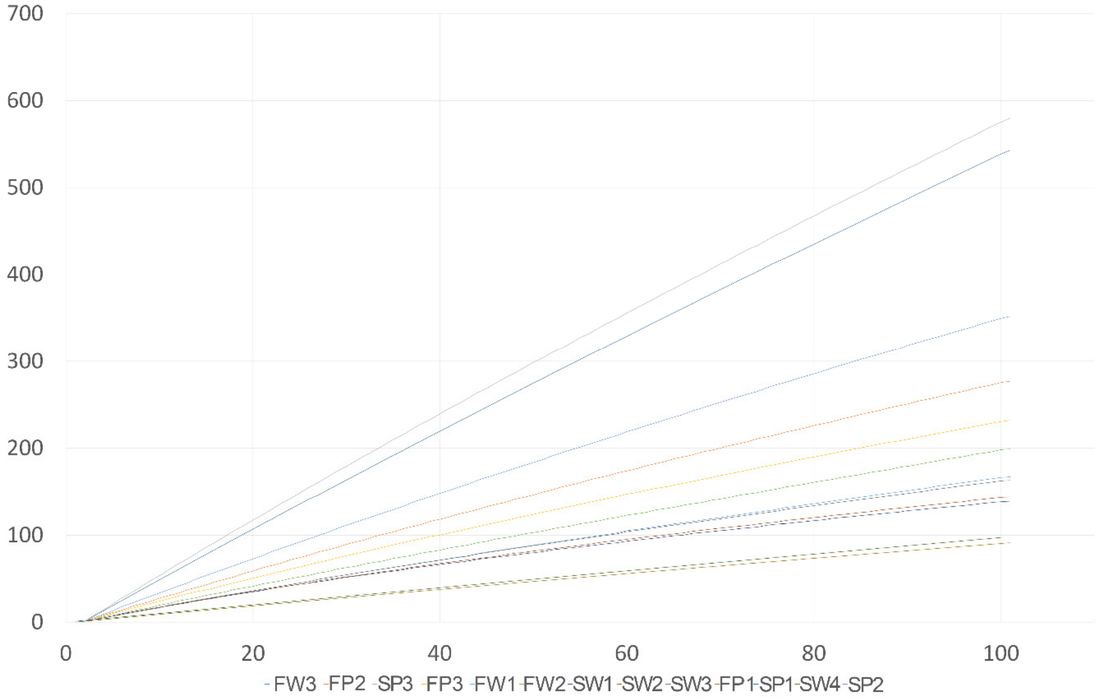

Figure 1. Estimation of rarefaction curves indicated a satisfactory level of diversity sampling.
